# ER stress inducer tunicamycin suppresses the self-renewal of glioma-initiating cell partly through inhibiting Sox2 translation

**DOI:** 10.18632/oncotarget.8954

**Published:** 2016-04-23

**Authors:** Yang Xing, Yuqing Ge, Chanjuan Liu, Xiaobiao Zhang, Jianhai Jiang, Yuanyan Wei

**Affiliations:** ^1^ Key Laboratory of Glycoconjuates Research, Ministry of Public Health, Department of Biochemistry and Molecular Biology, Shanghai Medical College of Fudan University, Shanghai, People's Republic of China; ^2^ Division of Neurosurgery, Zhongshan Hospital, Fudan University, Shanghai, People's Republic of China

**Keywords:** glioma-initiating cell, tunicamycin, Sox2, self-renewal

## Abstract

Glioma-initiating cells possess tumor-initiating potential and are relatively resistant to conventional chemotherapy and irradiation. Therefore, their elimination is an essential factor for the development of efficient therapy. Here, we report that endoplasmic reticulum (ER) stress inducer tunicamycin inhibits glioma-initiating cell self-renewal as determined by neurosphere formation assay. Moreover, tunicamycin decreases the efficiency of glioma-initiating cell to initiate tumor formation. Although tunicamycin induces glioma-initiating cell apoptosis, apoptosis inhibitor z-VAD-fmk only partly abrogates the reduction in glioma-initiating cell self-renewal induced by tunicamycin. Indeed, tunicamycin reduces the expression of self-renewal regulator Sox2 at translation level. Overexpression of Sox2 obviously abrogates the reduction in glioma-initiating cell self-renewal induced by tunicamycin. Taken together, tunicamycin suppresses the self-renewal and tumorigenic potential of glioma-initiating cell partly through reducing Sox2 translation. This finding provides a cue to potential effective treatment of glioblastoma through controlling stem cells.

## INTRODUCTION

Malignant gliomas remain the most lethal human brain tumors [1, 2]. Increasing evidence reveal that glioma-initiating cell (GIC) is responsible for the initiation, propagation, and recurrence of glioma [3–9]. Glioma-initiating cells are resistant to chemotherapy [10, 11]. Therefore, elimination of glioma-initiating cells is an essential factor for the development of efficient therapy.

The endoplasmic reticulum (ER) has emerged as a major site of cellular homeostasis regulation, particularly in the unfolded protein response (UPR) [12]. The significance of the ER-dependent pathways to cancer development also extends to clinical applications [13, 14]. Several anticancer agents also generate survival responses through activation of the unfolded protein response [15].

Tunicamycin, an N-glycosylation inhibitor, causes unfolded protein response and is widely used as pharmacological inducer of endoplasmic reticulum stress [16]. Several groups have reported that tunicamycin inhibits tumor cell growth and angiogenesis and enhances tumor cell apoptosis [17–19], offering a possibility for developing a new drug regimen for treating cancer. However, to date, few studies have addressed the role of ER stress in the homeostasis of tumor stem cells. These findings promote us to examine whether ER stress inhibits glioma-initiating cell self-renewal.

## RESULTS

### ER stress inducer tunicamycin decreases the self-renewal of glioma-initiating cell

**Figure 1:**
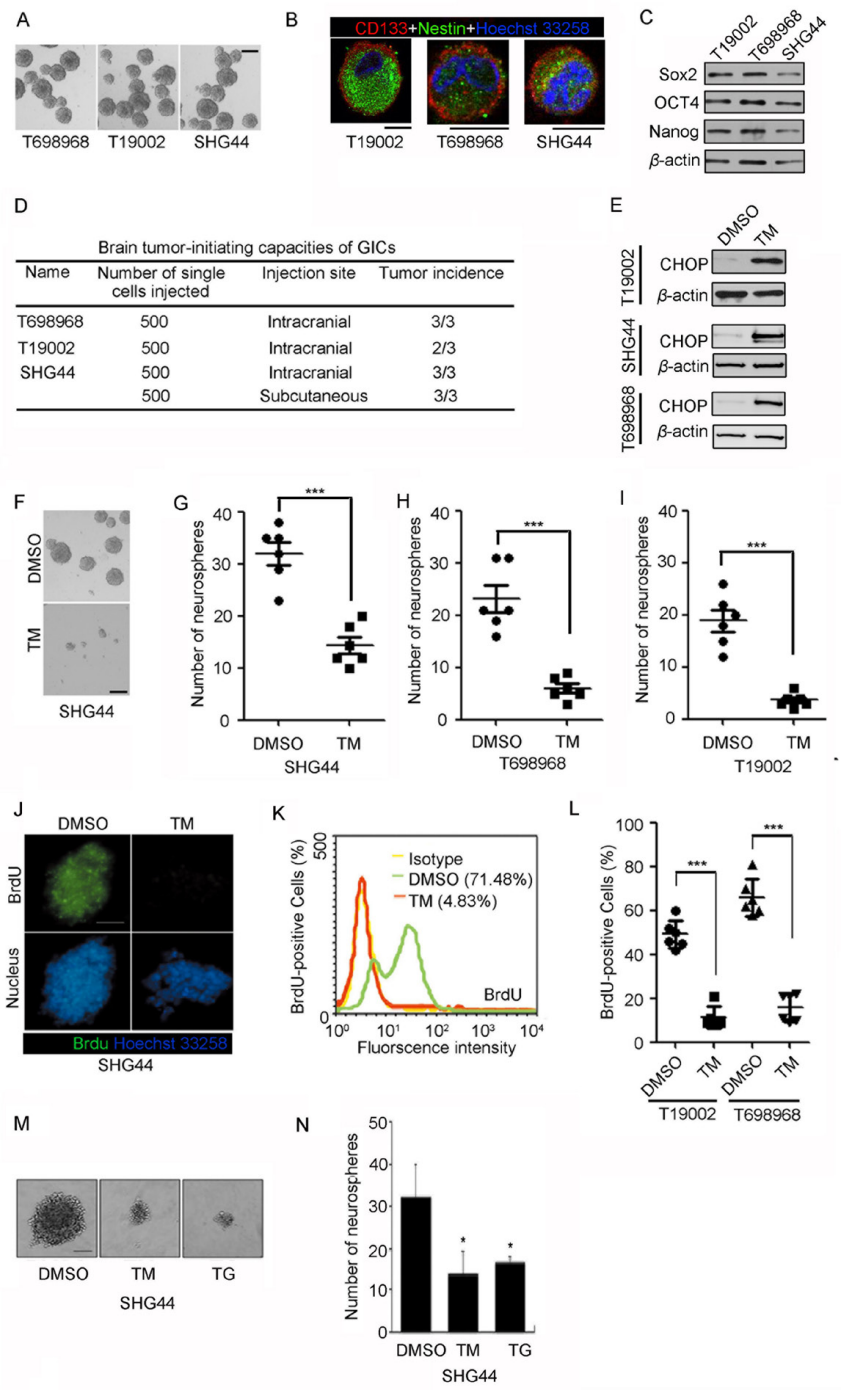
Tunicamycin inhibits the self-renewal of glioma-initiating cell (GIC) **A.** Representative images of neurospheres isolated from human GBM samples (T698968, T19002) and tumor xenograft formed by glioma cell line (SHG44). Scale bar represents 100 μm. **B.** Glioma-initiating cells expressed neural stem cell marker CD133 (red) and Nestin (green), as assessed by immunofluorescence. Nuclei were stained with Hoechst 33258 (blue). Scale bar represents 10 μm. **C.** Glioma-initiating cells expressed core stemness factors Sox2, OCT4 and Nanog, as assessed by western blot. *β*-actin expression served as loading control. **D.** Summary of the tumor-initiating capacity of the neurosphere cultures derived from human GBM samples and glioma xenograft. **E-I.** GICs were plated at 200 cells per well in 96-well plates in the presence of DMSO or 2.5 μM tunicamycin (TM) for seven days. Tunicamycin treatment increased expression of ER stress marker CHOP using western blot analysis (*E*). (*F*) Representative photographs of neurospheres formed by SHG44 GICs in the presence of DMSO or 2.5 μM tunicamycin (TM) for seven days. Scale bar represents 100 μm. (*G-I*) The numbers of neurospheres formed by SHG44 (*G*), T698968 (*H*) or T19002 GICs (*I*) in the presence of DMSO or 2.5 μM tunicamycin (TM) for seven days were determined. Values represent mean ± S.D. (n = 6, ***p < 0.001). **J-L.** BrdU incorporation assay in GICs treated with tunicamycin (TM). (*J*) Representative immunofluorescence images of BrdU incorporation in neurospheres formed by SHG44 GICs treated with DMSO or 2.5 μM tunicamycin (TM) for seven days (bars = 100 μm). (*K*) The percentage of BrdU-positive cells in SHG44 GICs treated with DMSO or 2.5 μM tunicamycin (TM) for seven days was determined by flow cytometry analysis. (*L*) The percentage of BrdU-positive cells in T19002 or T698968 GICs treated with DMSO or 2.5 μM tunicamycin (TM) for seven days was determined by flow cytometry analysis. Values represent mean ± S.D. (n = 6, ***p < 0.001). **M-N.** SHG44 GICs were plated at 200 cells per well in 96-well plates in the presence of DMSO or 2.5 μM tunicamycin (TM) or 2.5 μM thapsigargin (TG) for seven days. (*M*) Representative photographs of neurospheres formed by SHG44 GICs in the presence of DMSO or tunicamycin (TM) or thapsigargin (TG) for seven days. Scale bar represents 100 μm. (N) The numbers of neurospheres formed by SHG44 GIC in the presence of DMSO or tunicamycin (TM) or thapsigargin (TG) for seven days were determined. Values represent mean ± S.D. (n = 6, ***p < 0.001).

To examine whether ER stress inhibits glioma-initiating cell self-renewal, we generated primary cultures of neurospheres from human GBM samples (T698968, T19002) and glioma xenograft formed by glioma cell line (SHG44). The neurospheres showed characteristics consistent with GICs: namely, neurosphere formation (Figure 1A), expression of neural and/or cancer stem cell markers CD133 and Nestin (Figure 1B) and expression of core stemness factors Sox2, OCT4 and Nanog (Figure 1C). In tumor formation assay, as few as 500 cells were sufficient for tumor development in nude mice (Figure 1D).

The single cell neurosphere formation assay method is widely used to examine self-renewal potential of glioma-initiating cell [6, 22]. Tunicamycin (TM) significantly induced the expression of ER stress marker CHOP (Figure 1E), and reduced the number of newly formed neurospheres (Figure 1F–1I). Thus, tunicamycin reduces GICs self-renewal. Next, 5-Bromo-2-deoxyuridine (BrdU) incorporation assay was performed to further examine whether tunicamycin inhibited the self-renewal of GICs. The basal fraction of BrdU-positive cells of SHG44 GICs was 71.84% and decreased to 4.83% after tunicamycin treatment (Figure 1J and Figure 1K). Similarly, the rates of Brdu incorporation in T698968 and T19002 GICs were obviously reduced by tunicamycin (Figure 1L). Another ER stress inducer thapsigargin also reduced the number of neurospheres and decreased the diameter of neurospheres (Figure 1M and Figure 1N). Thus, tunicamycin decreases the self-renewal of GICs.

### Tunicamycin inhibits the tumorigenic potential of GICs

**Figure 2:**
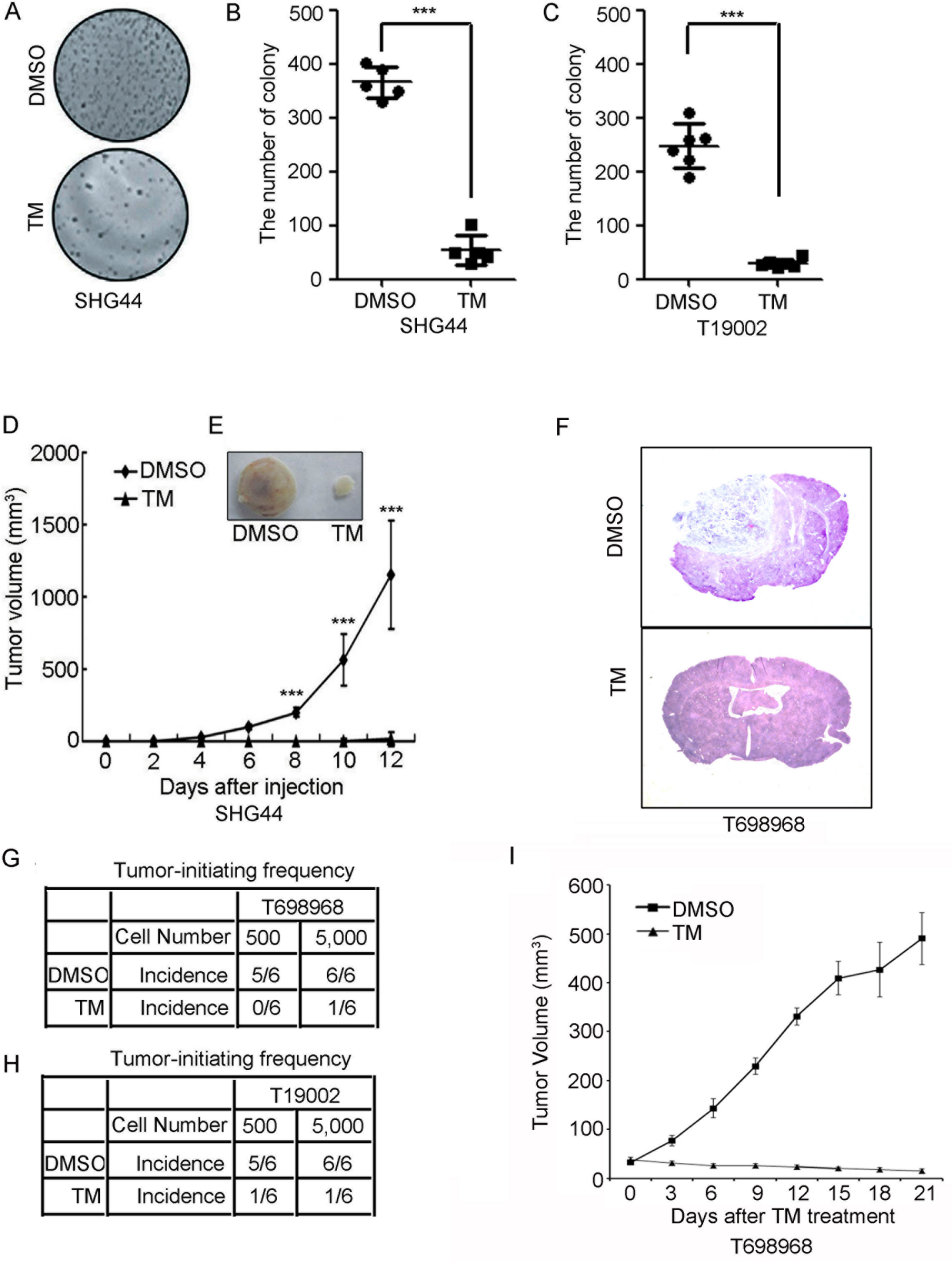
Tunicamycin inhibits the tumorigenic potential of GICs **A-C.** Tunicamycin reduced the number of colony formed by GICs. (*A*) Representative images of colony formed by 1, 000 viable SHG44 GICs pretreated with DMSO or 2.5 μM tunicamycin (TM) for 48 hours are shown. (*B-C*) The number of colony formed by 1, 000 SHG44 GICs (*B*) or T19002 GICs (*C*) pretreated with DMSO or 2.5 μM tunicamycin (TM) for 48 hours were counted. Values represent mean ± S.D. from three experiments (n = 6, ***p < 0.001). **D.** Nude mice were subcutaneously injected with 1 × 10^6^ SHG44 GICs cells treated with DMSO or tunicamycin (TM). Tumor volumes were measured every two days. Each point represents the mean volume ± S.D. of four tumors (***p < 0.001). **E.** After 6 weeks, nude mice were sacrificed and the dissected tumors were displayed. **F-H.** An intracranial limiting dilution tumor formation assay (employing 5,000 or 500 cells per mouse) was performed using DMSO- or tunicamycin (TM)-treated T698968 (*F-G*) or T19002 GICs (*H*). Mice were sacrificed when they were maintained up to 120 days or moribund after implantation. The table displays number of mice developing tumors (n = 6). **I.** Tunicamycin reduced the growth of GIC-initiated xenografts. Nude mice were subcutaneously injected with 1 × 10^6^ T698968 GICs cells. Tunicamycin treatment was started 7 days after the tumor development and was given orally. Tumor volumes were measured every three days. Each point represents the mean volume ± S.D. of six tumors.

We next examined whether tunicamycin inhibited the tumorigenic potential of GICs. Colony formation assay showed that tunicamycin substantially reduced the number of colony formed by SHG44 GICs (Figure 2A and 2B) and by T19002 GICs (Figure 2C). The reduction in the tumor formation of glioma-initiating cells by tunicamycin was further evaluated by tumor formation assay. TM-treated SHG44 GICs generated tumors with lower growth rate (Figure 2D), resulting in roughly 80-fold smaller tumor volume compared with DMSO-treated cells (Figure 2E). We further found that tunicamycin reduced the tumor-initiating potential of T698968 and T19002 GICs using intracranial tumor formation assay (Figure 2F–2H). These findings raised the question of whether tunicamycin could treat GIC-initiated xenografts. To address this point, tunicamycin treatment was started 7 days after the tumor development by T698968 cells. Compared to vesicle, tunicamycin obviously reduced the rate of tumor growth (Figure 2I). Thus, tunicamycin decreases the ability of GICs to initiate tumor formation.

### Inhibition of apoptosis partly abrogates the reduction in GIC self-renewal induced by tunicamycin

**Figure 3:**
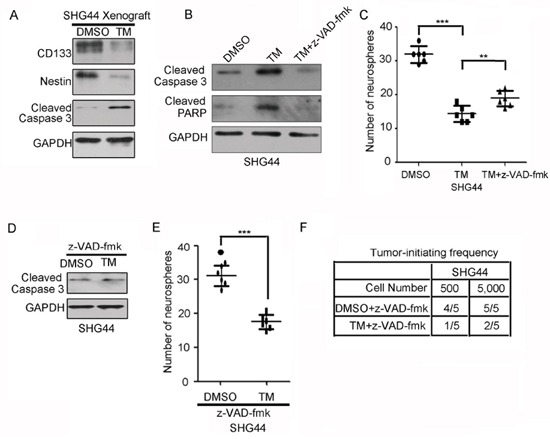
Inhibition of apoptosis partly abrogates the reduction in GIC self-renewal induced by tunicamycin **A.** Western blot analysis of stem cell markers CD133 and Nestin and apoptosis marker cleaved caspase 3 in subcutaneous xenograft formed by DMSO- and TM-treated SHG44 GICs. The expression of GAPDH served as a loading control. **B.** Western blot analysis of apoptosis marker cleaved caspase 3 and cleaved PARP in SHG44 GICs treated with or without the general caspase inhibitor z-VAD-fmk for 1 h before adding tunicamycin. The expression of GAPDH served as a loading control. **C.** The numbers of neurospheres formed by SHG44 GICs treated with or without tunicamycin (TM) and/or z-VAD-fmk for seven days were determined. Values represent mean ± S.D. (n = 6, ***p < 0.001). **D.** Western blot analysis of apoptosis marker cleaved caspase 3 in SHG44 GICs cells treated with the general caspase inhibitor z-VAD-fmk for 1 h before adding tunicamycin. **E.** The numbers of neurospheres formed by SHG44 GICs treated with z-VAD-fmk and/or tunicamycin (TM) for seven days were determined. Values represent mean ± S.D. (n = 6, ***p < 0.001). **F.** An intracranial tumor formation assay (employing 500, 5, 000 cells per mouse) was performed using SHG44 GICs treated with z-VAD-fmk and/or tunicamycin (TM). The table displays number of mice developing tumors (n = 5).

Tunicamycin has been reported to induce cell apoptosis [23]. Western blot analysis of resected xenografts formed by DMSO- and TM-treated SHG44 GICs showed that tunicamycin reduced the expression of stem cell markers CD133 and Nestin and increased the expression of apoptosis marker cleaved caspase-3 (Figure 3A). Thus, we first examined whether tunicamycin decreased the tumor-initiating ability of GICs resulting from cell death. To address this point, SHG44 GICs were treated with or without caspase general inhibitor z-VAD-fmk, followed by tunicamycin treatment. Tunicamycin increased the expression of apoptosis markers cleaved caspase-3 and cleaved PARP, indicating that tunicamycin induced GICs apoptosis. z-VAD-fmk significantly blocked activation of Caspase-3 cleavage and PARP cleavage by tunicamycin (Figure 3B). However, z-VAD-fmk only partly abrogated the reduction in glioma-initiating cell self-renewal induced by tunicamycin. (Figure 3C). Thus, inhibition of apoptosis did not completely abrogate the reduction in glioma-initiating cell self-renewal induced by tunicamycin. To further confirm this point, SHG44 GICs treated with z-VAD-fmk, were further treated with or without tunicamycin. Figure 3D showed that z-VAD-fmk significantly blocked activation of Caspase-3 cleavage by tunicamycin. However, tunicamycin still reduced the self-renewal and tumor-initiating potential of SHG44 GICs pretreated with z-VAD-fmk (Figure 3E and Figure 3F). Together, inhibition of apoptosis did not completely abrogate the reduction in GIC self-renewal induced by tunicamycin.

### Tunicamycin reduces the expression of self-renewal regulator Sox2

**Figure 4:**
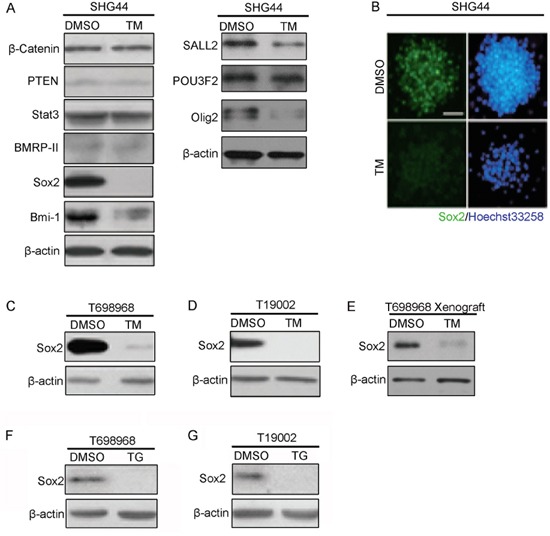
Tunicamycin inhibits the expression of transcription factor Sox2 **A.** Equal amounts of proteins from SHG44 GICs treated with DMSO or 2.5 μM tunicamycin (TM) for 48 hours were immunoblotted with the indicated antibodies. β-actin expression served as loading control. **B.** Expression of Sox2 (green) in SHG44 GICs cells treated with DMSO or tunicamycin (TM) was analyzed by immunofluorescence assay. Nuclei were stained with Hoechst 33258 (blue). **C-D.** Expression of Sox2 in T698968 (*C*) or T19002 GICs (*D*) cells treated with DMSO or tunicamycin (TM) was analyzed by western blot assay. β-actin expression served as loading control. **E.** Western blot analysis of Sox2 expression in subcutaneous xenograft formed by DMSO- and TM-treated T698968 GICs. The expression of β-actin served as a loading control. **F-G.** Expression of Sox2 in T698968 (*F*) or T19002 GICs (*G*) cells treated with DMSO or thapsigargin (TG) was analyzed by western blot assay. β-actin expression served as loading control.

To further explore the mechanisms of tunicamycin reducing GICs self-renewal, we investigated whether tunicamycin reduced the expression of genes regulating the self-renewal of glioma-initiating cell [24, 25]. Western blot assay and immunofluorescence assay showed that tunicamycin obviously reduced the expression of Sox2 (Figure 4A and 4B), a key gene sustaining self-renewal of normal and cancer stem cell [26–28]. Treatment with tunicamycin slightly reduced the expression of Bmi-1 and Olig2 proteins (Figure 4A). Down-regulation of Sox2 expression by tunicamycin was also observed in T698968 and T19002 GICs (Figure 4C and 4D). Furthermore, western blot analysis of xenografts resected from nude mice treated with or without tunicamycin showed that tunicamycin reduced the expression of Sox2 *in vivo* (Figure 4E). Consistent with this, another ER stress inducer thapsigargin also significantly reduced the expression of Sox2 (Figure 4F and 4G). Together, tunicamycin reduces the expression of self-renewal regulator Sox2.

### Sox2 overexpression obviously abrogates the reduction in GIC self-renewal induced by tunicamycin

**Figure 5:**
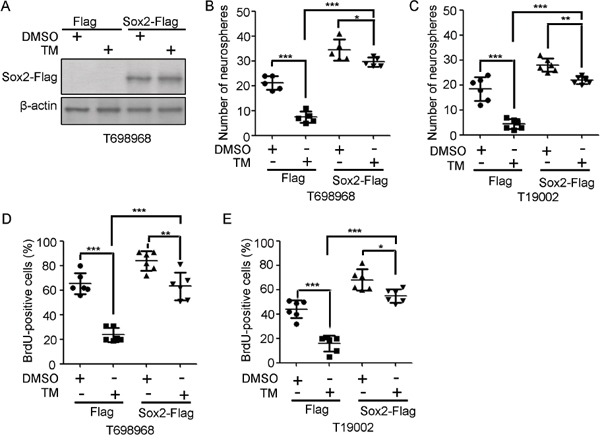
Sox2 overexpression partly abrogates the reduction in GIC self-renewal induced by tunicamycin **A.** Expression of exogenous Sox2 in T698968 GIC cells infected with LV-Flag or LV-Sox2-Flag lentivirus in response to tunicamycin treatment were examined by western blotting using Flag antibody. *β*-actin expression served as loading control. **B-C.** The numbers of neurospheres formed by T698968 (*B*) or T19002 GICs (*C*) infected with LV-Flag or LV-Sox2-Flag lentivirus in response to tunicamycin treatment for seven days were determined. Values represent mean ± S.D. (n = 6, ***p < 0.001, **p < 0.01, *p < 0.05). **D-E.** The percentage of BrdU-positive cells in T698968 (*D*) or T19002 GICs (*E*) infected with LV-Flag or LV-Sox2-Flag lentivirus treated with DMSO or tunicamycin (TM) for seven days was determined by flow cytometry analysis. Values represent mean ± S.D. (n = 6, ***p < 0.001, **p < 0.01, *p < 0.05).

Considering that Sox2 sustains GICs self-renewal [26, 27], we hypothesize that tunicamycin reduced GICs self-renewal partly by reducing Sox2 expression. To verify this hypothesis, GICs were infected with lentivirus expressing Flag or Flag-tagged Sox2 (Figure 5A). Sox2 over-expression increased the number of newly formed neurospheres by GICs and abolished the inhibitory effect of tunicamycin on neurospheres formation (Figure 5B and 5C). Consistent with this, Sox2 over-expression increased BrdU incorporation and abrogated tunicamycin-reduced BrdU incorporation (Figure 5D and 5E). Thus, tunicamycin inhibits the self-renewal of GICs at least partly through down-regulation of Sox2 expression.

### Tunicamycin reduces Sox2 expression at translation level

**Figure 6:**
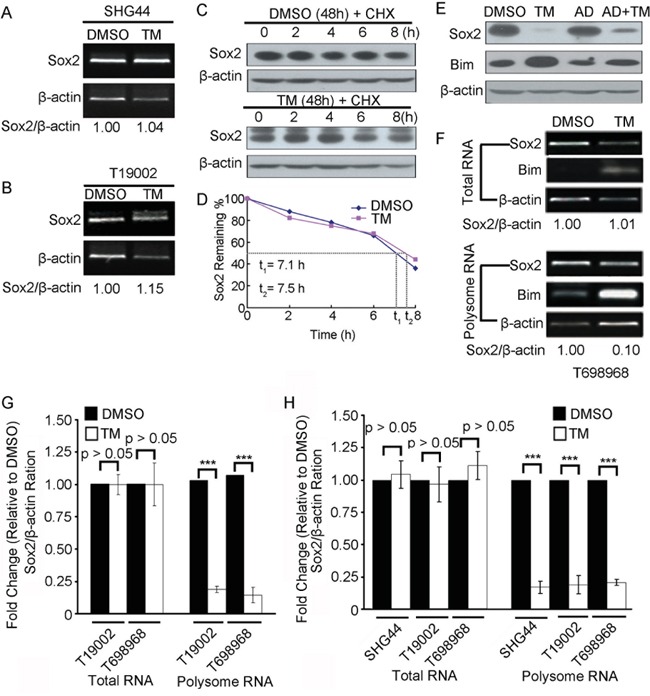
Tunicamycin reduces Sox2 expression at translation level **A-B.** RT-PCR analysis of Sox2 mRNA expression in SHG44 (*A*) or T19002 (*B*) GICs treated with DMSO or tunicamycin (TM). β-actin mRNA expression served as loading control. The ratio of Sox2 mRNA to β-actin mRNA was indicated. **C-D.** The stability of Sox2 protein in T698968 cells treated with DMSO or tunicamycin (TM). T698968 GICs were untreated or treated with 2.5 μM tunicamycin (TM) for 24 h, followed by a 100 μg/ml cycloheximide chase for the indicated time. (*C*) The representative figures are presented out. (*D*) Sox2 protein expression levels were normalized to β-actin level using Image-Pro Plus software, and the ratio in cells treated with DMSO or tunicamycin (TM) plotted against the time is shown. **E.** The T698968 GICs were treated with 50 μM actinomycin D (AD) alone or in combination with TM (AD+TM). Immunoblot analysis was carried out to measure the levels of Sox2, Bim and β-actin. Bim expression was increased at transcriptional and posttranslational during ER stress and served as a positive control. **F-G.** RT-PCR analysis of Sox2, β-actin and Bim mRNA in total RNA or polysomal RNA in T698968 or T19002 cells treated with DMSO or TM. Expression of β-actin served as a loading control. (*F*) The representative figures were presented out of three separate experiments. (*G*) Sox2 mRNA expression level in total RNA or polysomal RNA were normalized to β-actin level using Image-Pro Plus software, and the ratio in ER-stressed cells relative to unstressed (DMSO) is shown. Values represent mean ± S.D. (n = 3, ***p < 0.001). **H.** Real-time PCR analysis of Sox2 and β-actin mRNA in total RNA or polysomal RNA in cells treated with DMSO or tunicamycin (TM). Sox2 mRNA expression level were normalized to β-actin. Values represent mean ± S.D. (n = 4, ***p < 0.001).

To investigate the mechanism of tunicamycin reducing Sox2 expression, Sox2 mRNA expression in GICs treated with DMSO or tunicamycin was first examined using RT-PCR assay. Tunicamycin did not significantly reduce the level of Sox2 mRNA (Figure 6A, Figure 6B and Figure 6F, upper panel). CHX chase experiments further showed that tunicamycin did not significantly reduce the stability of Sox2 protein (Figure 6C and Figure 6D). It is widely known that ER stress inhibits protein translation through PERK-dependent phosphorylation of translation initiation factor 2 eIF2α [12], raising the possibility that tunicamycin reduces Sox2 expression at translation level. Pretreatment with transcription inhibitor Actinomycin D (AD) did not block the reduction in Sox2 protein expression induced by tunicamycin (Figure 6E), suggesting that the down-regulation of Sox2 protein expression by tunicamycin might result from a decrease in the new protein synthesis. To test this hypothesis, we performed a polysomal analysis of the Sox2 message RNA (mRNA) to determine its rate of translation initiation. Tunicamycin reduced Sox2 mRNA in the polysome fraction using RT-PCR assay (Figure 6F and 6G) and real-time PCR assay (Figure 6H). Together, tunicamycin reduces Sox2 expression at translation level.

## DISCUSSION

Altered N-glycosylation during tumor progression promotes tumor cell growth and invasion [29, 30]. Thus, inhibiting the synthetic pathway for N-linked glycosylation represents a novel approach in the treatment of cancer. N-glycosylation synthesis inhibitor tunicamycin inhibited tumor cell growth, angiogenesis and enhanced tumor cell apoptosis [17–19, 31, 32]. In this study, we evaluated whether tunicamycin inhibited GICs self-renewal. Tunicamycin markedly inhibited the neurosphere formation of glioma-initiating cell. Importantly, tunicamycin decreased the efficiency of glioma-initiating cell to initiate tumor formation. Since glioma-initiating cell initiates tumor formation [4, 7, 33, 34], these findings indicate that tunicamycin may be useful for glioma therapy. However, for clinical application, it is important to know whether tunicamycin can be given safely without toxicity to various normal tissues including brain.

Tunicamycin has been widely reported to induce cell apoptosis [18, 19, 23]. We also found that tunicamycin induced GICs apoptosis. Treatment with apoptosis inhibitor z-VAD-fmk partly abrogated the reduction in GICs self-renewal induced by tunicamycin. Even so, tunicamycin still reduced the self-renewal and tumor-initiating potential of GICs cells pretreated with z-VAD-fmk. Thus, inhibition of apoptosis did not completely abrogate the reduction in glioma-initiating cell self-renewal induced by tunicamycin.

Interestingly, tunicamycin reduces the expression of self-renewal regulator Sox2. Transcription factor Sox2 is widely known to sustain the self-renewal of several stem cell types, including embryonic stem (ES) cells and neuronal stem cells [26, 27]. Takahashi et al. showed that Sox2 in conjunction with KLF4, Oct4 and c-Myc, could induce pluripotency in both mice and human somatic cells [35]. To date, Sox2 has been found to be expressed in a variable percentage of cells in several malignant tissues, including glioma [36–39]. Gangemi et al. showed that Sox2 silencing in glioblastoma tumor-initiating cells inhibited its proliferation and tumorigenic ability [28]. Xuefeng Yang et al. showed that knockdown of the Sox2 gene in LN229 GBM cells reduced cell proliferation and colony formation [40]. Thus, Sox2 promotes glioma development, indicating that Sox2 would be an ideal target for glioblastoma therapy. Our data demonstrate that tunicamycin decreases the expression of Sox2. Furthermore, Sox2 overexpression obviously abrogated the reduction in GICs self-renewal induced by tunicamycin. Sox2 has been reported to activate expression of other pluripotency transcription factor [41]. Thus, tunicamycin inhibits the self-renewal of glioma-initiating cells partly through reducing Sox2 expression.

Another interesting finding is that tunicamycin reduces Sox2 expression at translation level. Deregulation of translation promotes oncogenic transformation [42–44]. Increased cap-dependent mRNA translation rates are frequently observed in human cancers [44]. Tunicamycin treatment leads to phosphorylation of the eukaryotic initiation factor 2alpha (eIF2α), resulting in attenuation of mRNA translation [12]. In addition, tunicamycin treatment leads to inactivation of eukaryotic translation initiation factor 4E (eIF4E). eIF4E overexpression protected cells from tunicamycin-induced cell growth arrest [45]. Our published data has shown that cap binding protein eIF4E activated Sox2 translation in glioma stem-like cells [46]. Based on these finding, we presume that tunicamycin might reduce Sox2 translation through inactivation of translation regulator eIF2 or eIF4E, which should be further investigated.

In conclusion, tunicamycin suppresses the self-renewal and tumorigenic potential of glioma-initiating cell partly through down-regulation of Sox2 translation. This finding provides a cue to potential effective treatment of glioblastoma through controlling stem cells.

## MATERIALS AND METHODS

### Glioma-initiating cell isolation and culture

GICs were established by isolating neurosphere-forming cells from surgical specimens of human GBM or human glioma xenografts using a method described previously [20–22]. The study was approved by the Institutional Review Board of Zhongshan Hospital of Fudan University and informed consent was obtained from all patients. GICs were cultured as GBM neurospheres in Dulbecco's modified Eagle's and F12 media supplemented with B27 (Invitrogen), 2 μg/ml heparin (Sigma), 20 ng/ml EGF (Chemicon) and 20 ng/ml FGF-2 (Chemicon). Human embryonic kidney cell line 293T cells were grown in DMEM medium (Invitrogen) supplemented with 10% fetal bovine serum in a 37°C incubator containing 5% CO_2_.

### Antibodies

The antibodies used were as follows: mouse anti-β-catenin antibody, mouse anti-Stat3 antibody and mouse anti-BrdU antibody were from BD Biosciences; mouse anti-Bmi-1 antibody and rabbit anti-OLIG2 and rabbit anti-POU3F2 was from Abcam; mouse anti-PTEN antibody and rabbit anti-CHOP antibody were from Cell Signaling Technology; mouse anti-Nestin antibody and rabbit anti-Sox2 antibody were from Millipore, mouse monoclonal anti-CD133 (W6B3C1 clone) was from Miltenyi Biotec.

### Polysome fractionation

Cells were treated with 100 μg/mL of cycloheximide 5 min before harvesting. One 100-mm dish of cells was scraped into 0.5 mL of lysis buffer (20 mM Hepes KOH at pH 7.2, 10 mM NaCl, 3 mM MgCl_2_, 0.5% NP40, 100 μg/mL cycloheximide, 200 U RNasin, and 1 tablet protease inhibitor per 10 mL). After 15 min incubation on ice, lysates were transferred to a 1 mL dounce homogenizer on ice and cells lysed with 10 strokes. The nuclei were pelleted in a microcentrifuge at 3, 000 × g for 2 min. The supernatant was transferred to a fresh tube and 500 μg/ml heparin was added. Ribosome components were separated from the soluble fraction by centrifugation at ~ 430,000 × g for 25 min at 4°C using a SW60Ti rotor in a Beckman ultracentrifuge. The ribosome pellet was re-suspended in lysis buffer. RNA was isolated using Trizol (Invitrogen). Levels of mRNA were analyzed using RT-PCR or real time-PCR.

### Lentivirus production and GICs infection

For ectopic expression of human Sox2, LV-Sox2-Flag plasmid was constructed by inserting full length human Sox2 cDNA into the LV-Flag lentivirus vector between BamHI and AgeI sites. The lentiviral vectors were co-transfected with the packaging vectors into 293T cells by the calcium phosphate co-precipitation method to produce virus. Two days following transfection, viral supernatants were collected, filtered, and concentrated by ultracentrifugation. Neurospheres were dissociated and resuspended in growth media, mixed with virus and plated. Polybrene was added at a final concentration of 8 μg/ml. Cells were incubated with virus for 12 hours, washed with PBS, and incubated in fresh media.

### Neurosphere formation assay

An equal number of cells were seeded at low cell density (200 cells/well) in a 96-well plate. The total number of newly formed neurospheres was counted after 7 days in culture. Spheres that contained more than 20 cells were scored.

### Colony formation assay

The colony formation assay was carried out in 35 mm dishes. Briefly, cells were plated in 35 mm dishes at 1,000 cells/well in 0.35% top agar in culture medium over a 0.5% agar layer. For compound testing group, compounds were added into the top agar at concentrations as indicated. Plates were further incubated in cell culture incubator for 12 days until colonies were large enough to be visualized. Colonies were stained with 0.01% Crystal Violet for 1 h and counted.

### Proliferation assay

Cells were given a 24 hr pulse of BrdU (Sigma) at 30 μg/ml. Visualization of new DNA synthesis was revealed by anti-BrdU indirect immunofluorescence. For BrdU immunofluorescence, cells were treated and post-fixed in 4% paraformaldehyde in PBS for 30 min at 4°C, rinsed in 0.1 M PBS (pH 7.4) with 1% Triton X-100, followed by incubation in 2 N HCl for 60 min at 37°C to open the DNA structure of the labeled cells, and further rinsed with 0.1 M borate buffer (pH 8.5) for 12 min at room temperature. After washing for 5 min in PBS with 1% Triton X-100, samples were incubated in 0.1 M PBS (containing 1% Triton X-100, 1 M glycine, and 5% normal goat serum) for 1 hr prior to incubating overnight at 4°C with mouse anti-BrdU antibody. This was followed by washing in 0.1 M PBS (pH 7.4) (containing 1% Triton X-100) for 5 min, and cells were next incubated with FITC-conjugated goat anti-mouse IgG for 2 hr at 37°C. Analysis was performed by microscopy and FACS.

### Subcutaneous tumor formation assay

GICs were centrifuged and re-suspended in a sterile solution of PBS at a final concentration of about 1.0 × 10^7^ cells/ml. A 100 μl aliquot of re-suspended cells (about 1.0 × 10^6^ cells) was injected subcutaneously into the flanks of 6-week old athymic nude mice. Tumor measurements were done every two days using traceable digital vernier calipers. The tumor volumes were determined by measuring the length (l) and the width (w) and calculating the volume using the formula V = lw^2^/2. To examine whether tunicamycin could treat GIC-initiated xenografts, tunicamycin treatment was started 7 days after the tumor development. Mice were treated with tunicamycin (0.25 mg/kg) orally every three days.

### Intracranial tumor formation assay

Intracranial transplantation of GICs into nude mice was performed as previously described [20, 22], in accordance with a Fudan University Animal Care and Use Committee approved protocol concurrent with national regulatory standards. Briefly, cells were counted and certain number cells were injected intracranially into nude mice. Mice were maintained up to 16 weeks or until the development of neurological symptoms. Each mouse's brain was harvested, fixed in 4% formaldehyde and embedded in paraffin. Tumor formation was determined by histologic analysis of H&E-stained sections.

### Immunofluorescence

For immunostaining of undifferentiated tumor spheres, cells were fixed with 4% PFA for 20 minutes at room temperature, washed three times with PBS, and then blocked with a PBS based solution containing 5% normal serum and 0.3% Triton X-100. Cells were incubated overnight with rabbit polyclonal anti-Nestin or mouse anti-CD133 antibody. After washed three times with PBS, cells were incubated with goat anti-rabbit Alexa 488 IgG or goat anti-mouse Alexa 594 IgG. Nuclei were counterstained with Hoechst 33258 (Sigma; 10 μg/ml).

### Statistical analysis

For analysis of experimental data, comparison of categorical data was carried out by Student's *t*-test. Data are presented as the mean ± *S.D*. All P values are two-sided. *p < 0.05 was considered statistically significant in all experiments.

## Abbreviations

GIC: glioma-initiating cell
GBM: glioblastoma multiforme
TM: tunicamycin
TG: thapsigargin
ECL: enhanced chemiluminescence
RT: reverse transcription
Ab: antibody
